# Cryogenic Investigations into the Effect of Impact-Oscillatory Loading on Changes in the Mechanical Properties and Structural Condition of VT23M Two-Phase Titanium Alloy

**DOI:** 10.3390/ma17163913

**Published:** 2024-08-07

**Authors:** Mykola Chausov, Volodymyr Hutsaylyuk, Pavlo Maruschak, Andrii Pylypenko, Myroslav Karpets, Vladyslav Shmanenko

**Affiliations:** 1 Department of Mechanics, National University of Life and Environmental Sciences of Ukraine, Heroiv Oborony Str. 15, 03041 Kyiv, Ukraine; chausov@nubip.edu.ua (M.C.); andriy3pl@gmail.com (A.P.); vladshmanenko@ukr.net (V.S.); 2 Faculty of Mechanical Engineering, Military University of Technology, Gen. S. Kaliskiego Str. 2, 00-908 Warsaw, Poland; 3 Department of Industrial Automation, Ternopil National Ivan Puluj Technical University, Ruska Str. 56, 46001 Ternopil, Ukraine; maruschak.tu.edu@gmail.com; 4 Department of Materials Science and Heat Treatment, National Technical University of Ukraine “Igor Sikorsky Kyiv Polytechnic Institute”, 37, Beresteisky Avenue, 03056 Kyiv, Ukraine; mkarpets@ukr.net; 5 V. Bakul Institute for Superhard Materials of the National Academy of Sciences of Ukraine, Avtozavodska Str., 2, Kyiv 04074, Ukraine

**Keywords:** titanium alloy, strength, plastic properties, impact-oscillatory loading

## Abstract

Impact-oscillatory loading of variable intensity was applied to the VT23M high-strength sheet two-phase titanium alloy in liquid nitrogen. This was carried out to investigate the effect of this loading type on changes in the mechanical properties and structural condition of the alloy upon subsequent static tensioning at normal temperature. Dynamic non-equilibrium process (DNP) realized at a temperature of liquid nitrogen proved to be unable to impair the strength properties of the VT23M titanium alloy compared to room temperature; however, they caused a significant decrease in ductility (down to 16%). The impaired plastic properties of the alloy were shown to entail additional defects in the structural components of the alloy. The authors have found patterns of damage accumulation in the structural components of the alloy depending on the DNP parameters. They are in good agreement with the findings of the fractographic research.

## 1. Introduction

The papers in [1,2,3,4,5,6,7,8,9,10,11,12,13] propose and put into practice the main approaches to enhancing the mechanical properties of high-strength (α + β) titanium alloys. In particular, the alloys were subjected to complex alloying conditions and special thermal and thermomechanical treatment conditions. As a result, two-phase titanium alloys with enhanced strength and plastic properties were obtained (σ_us_ not below 1100 MPa and δ up to 20%).

High-strength two-phase titanium alloys have been widely used in aircraft and rocket engineering. The VT23M two-phase high-strength titanium alloy appeared most promising in this regard. The VT23M alloy is a modification of the VT23 alloy—another well-known two-phase titanium alloy of the martensitic class. Compared to the VT23 alloy, the former has a narrow alloying range. This accounts for the improvements in the guaranteed level of mechanical properties.

Currently, the range of load-bearing structures made of the VT23M alloy is expanding. Apart from being used in structures operating at cryogenic temperatures, the VT23M alloy was shown to be promising in the production of structures operating in arctic climates [14].

In practice, most findings concerning the mechanical properties of the VT23M alloy at various temperatures were obtained under standard static tensioning conditions [15]. When operated, structures from the VT23M alloy may be subjected to more complex loading conditions, in particular impact-oscillatory loading. The paper in [16] describes the mechanical testing technique that uses impact-oscillatory loading of variable intensity. This technique can be easily realized on any hydraulic testing machine equipped with an additional internal unit, which is a statically indeterminate system. Here, it should be noted that any structural material, including titanium alloys, is affected by chaotic dynamics (dynamic non-equilibrium process (DNP) under such complex loading conditions. Along with that, the alloy structure formed during previous complex technological operations undergoes significant changes in a very short time (35...45 milliseconds). This affects the mechanical properties of titanium alloys, in particular upon repeated static tensioning. In this case, the reference mechanical properties are those formed during static tensioning of baseline materials at room temperature. They should be used to compare changes in the mechanical properties.

Based on many years of research, the authors recommend impact-oscillatory loading firstly as a simple technological method for improving the mechanical properties of titanium alloys in finished rolled products. To this end, the optimal level of DNP needs to be provided at room temperature. In turn, DNP may cause damage that significantly impairs the mechanical properties of alloys in operation [17,18]. Notably, sudden variations in the mechanical properties and structural parameters of materials upon DNP depend on the quality of strength calculations made for load-bearing structures. Most of them are statically indeterminate mechanical systems, in which the above processes may occur under impact-oscillatory loading. So far, few attempts have been made to improve the strength calculations for structural materials affected by DNPs [19].

Impact-oscillatory loading is complicated by:

- Cooling of titanium alloys in liquid nitrogen + DNP + static tensioning (ST);
- DNP + cooling in liquid nitrogen + ST.

This has revealed other interesting effects that cause significant changes in the mechanical properties of titanium alloys [20]. However, previous research does not deal with DNP in liquid nitrogen. Therefore, evaluating changes in the mechanical properties of titanium alloys appears complicated.

This paper aims to evaluate the effect of DNP in liquid nitrogen on changes in the mechanical and structural parameters of the VT23M alloy upon repeated static tensioning at room temperature.

## 2. Materials and Methods

The mechanical testing technique was realized on a modified ZD-100Pu hydraulic installation (WPM, Leipzig, Germany) for static testing and is described in detail in [11]. The proposed technique’s main idea consists of high-speed tensioning of the material with the imposition of a high frequency (1–2 kHz) oscillatory process, which corresponds to the natural frequency of the testing machine. Structurally, this is achieved by an inner contour introduced into the testing machine in addition to the outer contour (loaded frame of the testing machine). The inner contour is the simplest statically indeterminate structure in the form of three parallel elements loaded simultaneously—the central specimen and two satellites (brittle samples) of different cross-sections made of hardened steels 65G or U8–U12. When this structure is tensioned, the satellites are destroyed, and the energy is introduced into the specimen in a pulsed manner. As a result, DNP occurs in the specimen material. Satellites may get involved in the operation at any stage of preliminary static tensioning. This makes it possible to find out whether the impulse introduction of energy improves or worsens the mechanical properties of materials upon subsequent static tensioning. By changing the initial diameter of the satellites, it is possible to control the intensity of the impulse introduction of force energy into the material. 

**Figure 1:**
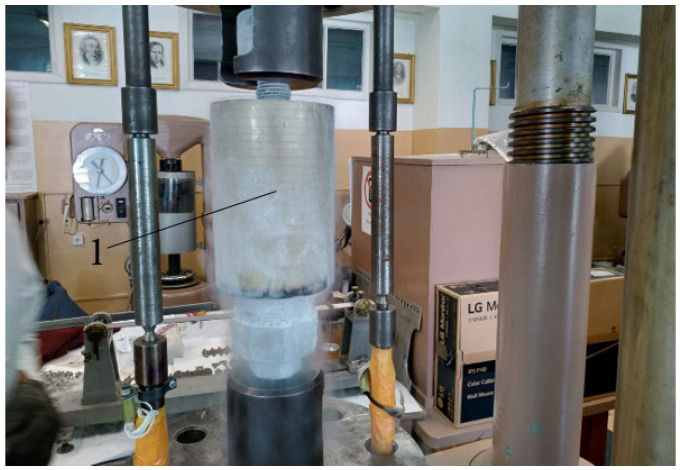
General view of the mechanical testing machine equipped with cooling chamber 1.

In this study, the device for introducing impulse energy was improved by adding a cooling chamber (see Figure 1) for impact-oscillatory loading in liquid nitrogen.

**Figure 2:**
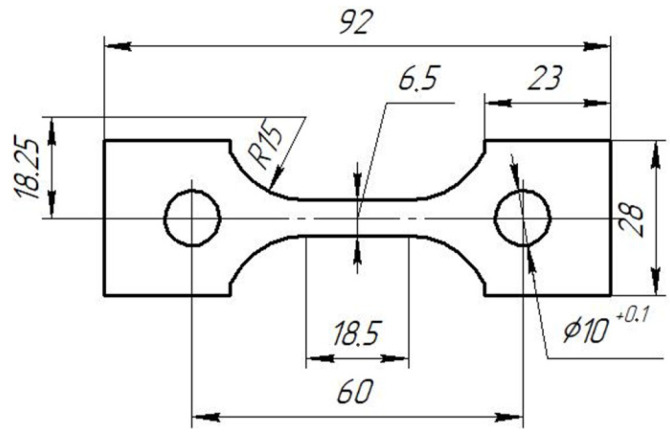
Specimen for mechanical tests.

Mechanical tests were performed on 3 mm thick specimens (Figure 2) from the VT23M sheet titanium alloy.

As in previous papers, DNP parameters in the alloy were controlled by sudden increases in dynamic strain *ε_imp_* under DNP [17]. To simplify the test procedure, *ε_imp_* was chosen as a parameter that characterizes the intensity of introducing impulse energy into alloys. This procedure was also used to evaluate further deformation of materials under DNP. 

Specimens subjected to impact-oscillatory loading were used for *ε_imp_* measurements using the Optika B-510 MET microscope (New York Microscope Company, New York, NY, USA). The initial measurement base was 16 mm. Measurement accuracy was up to 0.002 mm. Upon subsequent static tensioning, a standard 0.5 accuracy class extensometer manufactured at the Antonov aircraft production plant, Kyiv, Ukraine, was used for strain measurements.

The percentage composition of α and β phases in the VT23M titanium alloy at baseline and after DNP in liquid nitrogen was evaluated radiographically. Monochromatic Cu Kα radiation generated by the Ultima IV diffractometer (Rigaku, Japan) was used for X-ray investigations conducted on 10 mm long specimen sections.

Fractographic studies of specimen fractures to detect morphological differences in the fracture micromechanisms were performed on the Zeiss EVO-40XVp (ZEISS International, Oberkochen, Germany) scanning electron microscope. 

The shape and size of dimples of ductile tearing formed during static and impact fracture of titanium alloy VT 23M were analyzed by an automated method. The method is based on the analysis of the image topology. The method contains the operations of smoothing the initial fractographic image; convolution with a filter to identify topological ridges; thresholding with subsequent skeletonization to identify boundaries between dimples; clustering to isolate the connected areas that represent the objects of interest, i.e., the dimples. For each dimple, the following quantitative characteristic was calculated: dimple equivalent diameter Deq. [21,22].

The mechanical properties of the baseline VT23M titanium alloy are given in Table 1.

The chemical composition of the VT23M alloy is given in Table 2.

## 3. Results and Discussion

### 3.1. Mechanical Tests

The paper in [17] presents an investigation partially dealing with the mechanical properties of the VT23M titanium alloy (the chemical composition and mechanical properties of the alloy are described above). The alloy was first subjected to DNP at room temperature (the intensity of introducing impulse energy varied in a wide range) and then to static tensioning also at room temperature. 

A batch of 11 specimens was tested in this research. Specimens 1–10 were previously tested in liquid nitrogen. Specimen 11 was statically deformed to failure at room temperature. The paper in [17] presents stress–strains diagrams obtained for this alloy at room temperature in accordance with the current standard. Given this, a decision was made to conduct a control test of the specimen at room temperature to compare the results obtained.

In this research, impact-oscillatory loading was pre-applied to the VT23M alloy in liquid nitrogen as follows: first, all specimens were loaded statically to a stress level of 50 MPa and then they were subjected to DNP. As a result, some specimens subjected to DNP retained plastic deformation of various levels and were then unloaded. Other specimens remained in the plastic region until residual strain *ε_imp_* = 0.57% was attained after unloading. 

Prior to DNP, specimens were held in liquid nitrogen for 1 h as described in the paper in [16]. Unfortunately, with a further increase in the DNP intensity (*ε_imp_* > 0.6%), specimens were dynamically destroyed in liquid nitrogen, making it impossible to find the critical value of *ε_imp_* (the intensity of introducing impulse energy into the alloy of interest). According to some rough results, this value does not exceed 1.0%.

**Figure 3:**
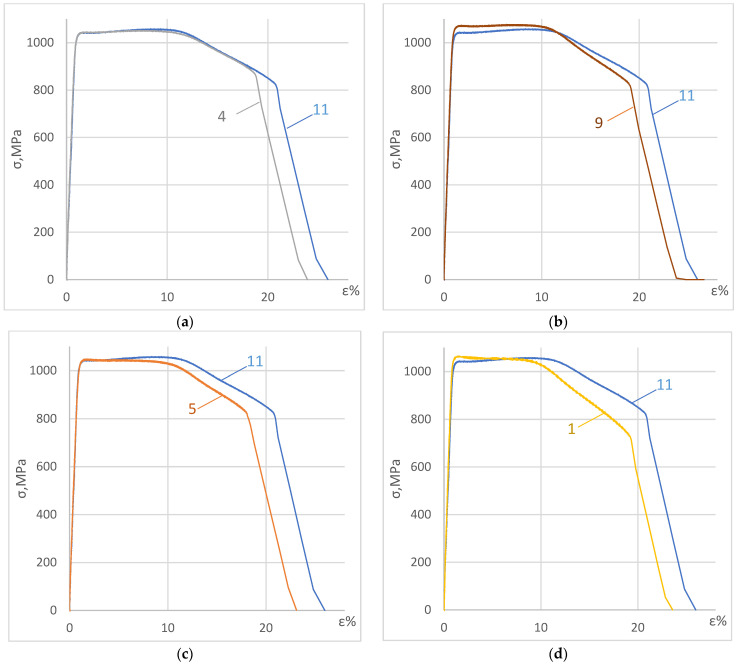
Stress–strain diagrams (**a**–**d**) obtained for the VT23M alloy: 11—in the initial state at room temperature, 4, 9, 5, 1—upon DNP in liquid nitrogen at *ε_imp_* = 0.12; 0.22; 0.28; 0.53%. The numbers of the test specimens are indicated on the curves.

**Figure 4:**
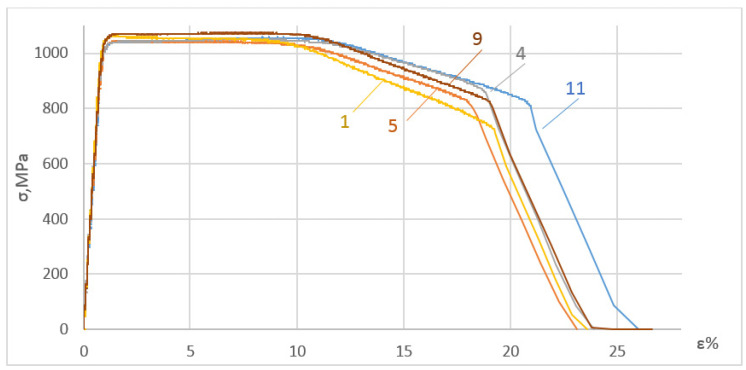
Summary of stress–strain diagrams obtained for the VT23M alloy (designations of the curves correspond to those in Figure 3).

Figure 3 presents some test results obtained for the specimens that were first subjected to DNP in liquid nitrogen and then statically tensioned at room temperature. For clarity of the data obtained, each stress–strain diagram obtained under such complex loading conditions was compared to the ST diagram of the baseline VT23M alloy statically tensioned at room temperature (Figure 3a–d). Figure 4 presents stress–strain diagrams versus the ST diagrams.

The research findings indicate that DNP in liquid nitrogen changes the mechanical properties of the VT23M alloy upon subsequent static tensioning, as evidenced by the elastic-plastic branch of the stress–strain diagram. On the elastic branch of the stress–strain diagram, DNP had practically no effect on the mechanical properties of the VT23M alloy (see Figure 3a).

Damage accumulated more intensively when the alloy was deformed plastically during DNP. As a result, the kink on the descending branch of the stress–strain diagram (compare Figure 3c,d) has changed. The paper in [19] presents a detailed analysis of the gradual nature of damage accumulation in the alloy. The parameters of the descending branches of the complete stress–strain diagrams were key to the analysis. Thus, DNP in liquid nitrogen changes damage accumulation kinetics in the VT23M alloy and, accordingly, reduces its overall deformation. The baseline alloy was damaged during DNP in liquid nitrogen. As a result, the strain level was reduced to 12% upon repeated static tensioning.

### 3.2. Diffractometric Research Findings

**Figure 5:**
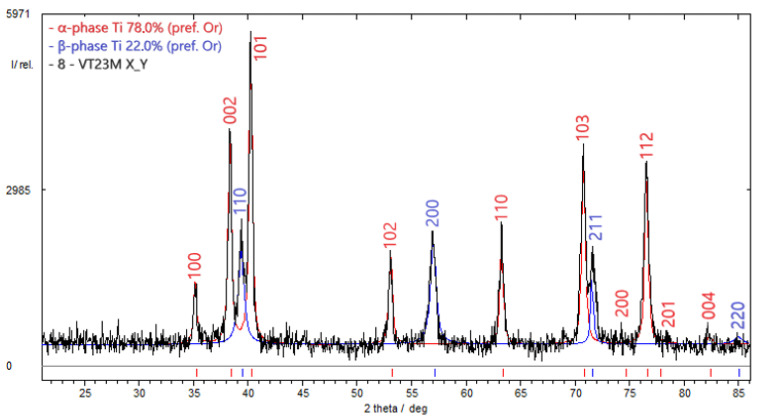
Results of the diffractometric study to evaluate the percentage of α and β phases in the titanium alloy VT23M in the initial state: α-phase occupied 78% by weight, β-phase—22%.

The diffractometric research findings (Figure 5) indicate that β-phase occupies 22% by weight and α-phase—78% by weight in the VT23M titanium alloy at baseline. The investigation was conducted in the central part of the 10 mm long section of an undeformed specimen. Characteristically, both phase components have a texture in the crystallographic direction (002). This may result from rolling or other mechanical treatment of the specimen. 

As noted above, the microstructure of the V23M alloy is damaged during DNP in liquid nitrogen, which has a significant effect on changes in the mechanical properties of the alloy upon subsequent ST (see Figure 3 and Figure 4).

Many methods have been proposed to evaluate the damage to materials during loading [23,24]. Each of them has its own advantages and disadvantages. Clearly, non-destructive methods need to be given priority. Earlier, the authors proposed a method for evaluating damage of materials under loading by changing the percentage composition of the main chemical elements or main phases in the material matrix [25]. According to this patent, the material damageability (D) under a certain loading type can be calculated from the following formula:

(1) $$D=\frac{\varphi_{init}-\varphi_{imp}}{\varphi_{init}}\cdot 100\%$$

where *φ_init_* is the percentage composition of the main chemical element or phase before loading, and *φ_imp_* is the percentage composition of the main chemical element or phase after loading.

Here, we do not refer to the actual changes in the percentage composition of the main chemical elements or phases in the material matrix under loading. Rather, we presume changes in the percentage composition of a certain main chemical element or phase. Such changes may be caused by damage accumulation in the main phases of the material, which can be manifested, for instance, in the course of diffractometric investigation. Thus, if major damage occurring in the alloy under DNP in liquid nitrogen is assumed to be more brittle than the α-phase, this makes it possible to predict an apparent decrease in the percentage composition of this particular phase. Subsequent diffractometric studies fully confirmed this assumption.

**Figure 6:**
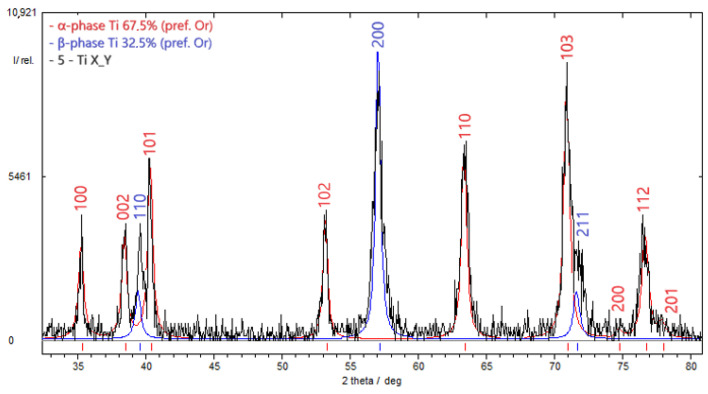
Results of the diffractometric study to evaluate the percentage of α and β phases in the titanium alloy VT23M pre-subjected to IOL with *ε_imp_* = 0.28%: α-phase occupied 67.5% by weight, β-phase was 32.5%.

**Figure 7:**
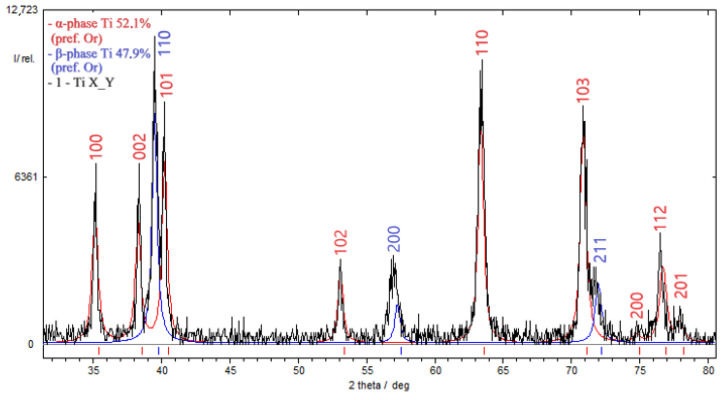
Results of the diffractometric study to evaluate the percentage of α and β phases in titanium alloy VT23M pre-subjected to IOL with *ε_imp_* = 0.53%: α-phase occupied 52.1% by weight, β-phase was 47.9%.

Figure 6 and Figure 7 present the results of diffractometric investigations into specimens from the VT23M alloy subjected to DNP in liquid nitrogen at *ε_imp_* = 0.28 and 0.53%, respectively, and then to static tensioning at room temperature. Diffractometric investigations were conducted on a 10 mm long section adjacent to the fracture surface. 

Based on the results of diffractometric research using Formula (1), the damageability of α-phase is D = 12.46% at *ε_imp_* = 0.28%, or D = 33.21% at *ε_imp_* = 0.53%. In other words, with an increase in the DNP intensity, the α-phase, which is more brittle, tends to accumulate more damage in the matrix of the VT23M alloy.

**Figure 8:**
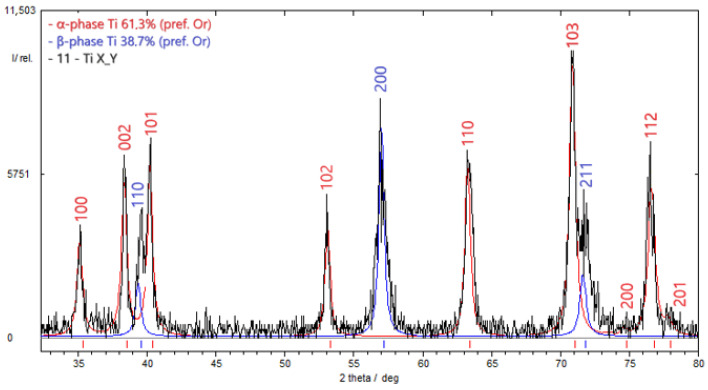
Results of the diffractometric study to evaluate the percentage of α and β phases in the titanium alloy VT23M at baseline, which was destroyed by static tensioning at room temperature: α-phase occupied 61.3% by weight, β-phase was 38.7%.

To confirm the reliability of the proposed method for evaluating damage based on the percentage composition of α-phase in the alloy, similar studies were conducted on a specimen from the alloy in the initial state, which was destroyed by ST at room temperature. The corresponding research findings are presented in (Figure 8). As seen in Figure 8, the percentage composition (%) of α-phase in the zone adjacent to the fracture surface has also decreased significantly.

Using Formula (1), we obtain D = 21.41%. Damageability of α-phase upon ST at room temperature in the fracture zone of the VT23M alloy is greater than that after DNP in liquid nitrogen at *ε_imp_* = 0.28%. To explain this, we analyzed the material surface with ultimate damage depending on the stress state type and duration of the previous service [19]. The surface is constructed according to the parameters indicated on the descending branches of the complete stress–strain diagrams. In other words, the metallophysical research findings make it possible to reliably determine the ultimate damage when a macrocrack is formed in the material depending on the stress state type and duration of previous service of the material. Investigations described in [26] indicate that each process characterized by its own deformation history has its own damage development curve and, accordingly, its own ultimate damage. This explains the results obtained. It should also be noted that the ultimate damage of the material is equivalent to the specific work of fracture. Therefore, the paper in [26] also shows how the parameters indicated on the descending branch of the complete stress–strain diagram can be used to determine the fracture toughness of the material depending on the stress state type and duration of previous service. This procedure was used to evaluate the effect of complex loading conditions on changes in the fracture toughness of titanium alloys [20].

### 3.3. Fractographic Research Findings

Fractographic analysis of fracture surfaces of specimens made of the VT23M titanium alloy pre-subjected to different loading conditions was performed to explain the effects of damage caused to the VT23M alloy structure subjected to DNP in liquid nitrogen.

Fractures of specimens 11, 9, 5, and 1, the stress–strain diagrams of which differ significantly, were selected for the analysis (see Figure 3 and Figure 4). For comparison, the fractographic investigation into the specimen fracture, which was destroyed dynamically upon DNP in liquid nitrogen (specimen 7), was conducted. The fracture analysis of the fracture surfaces of these specimens testifies to significant differences in their morphology.

**Figure 9:**
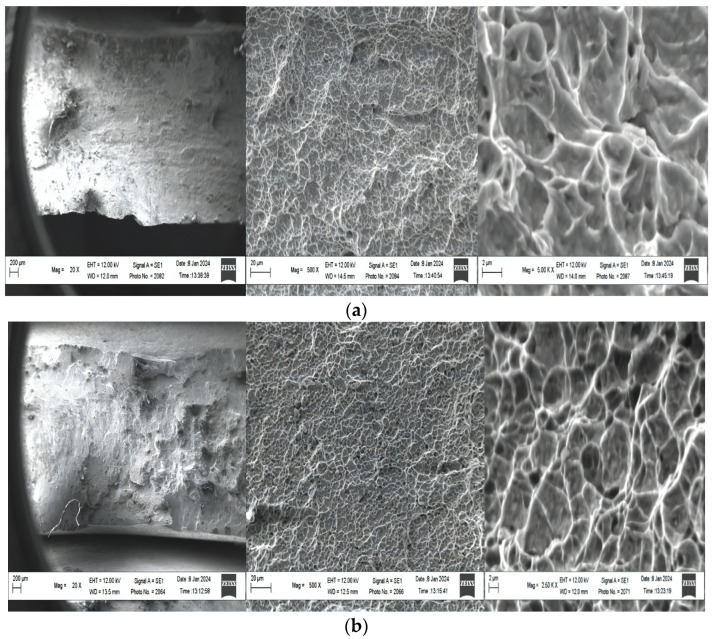

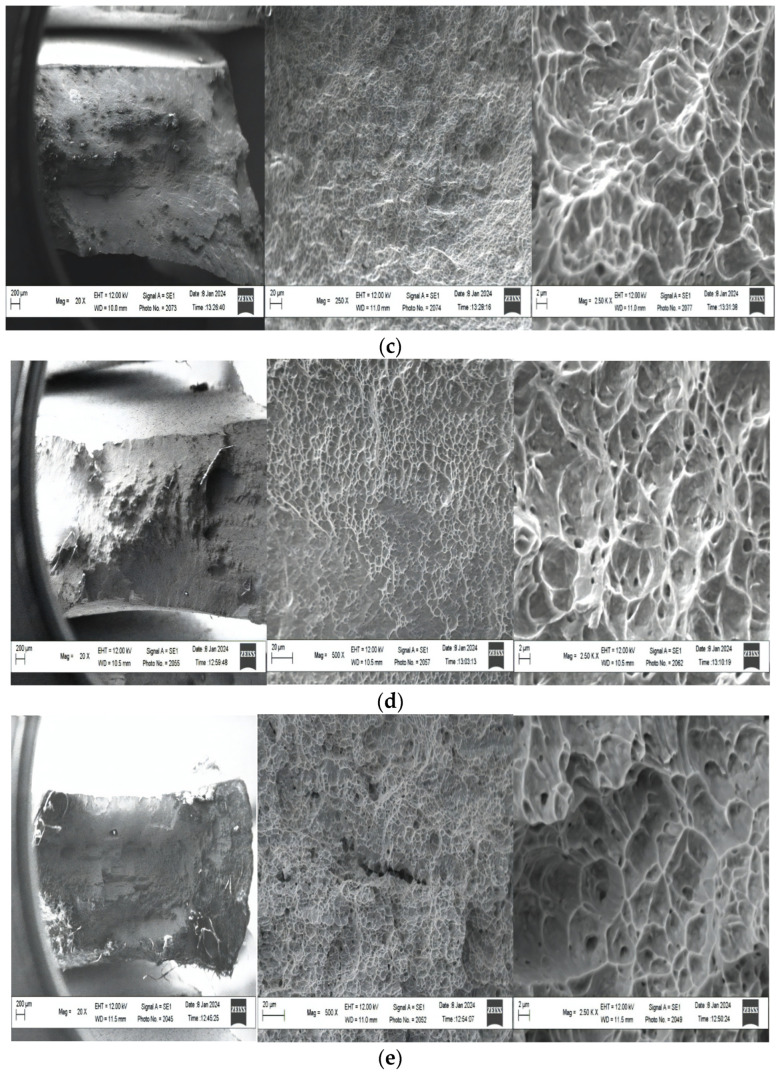
Fractures of specimens made of the VT23M titanium alloy subjected to different loading conditions (designations (**a**,**c**–**e**) correspond to specimens 11, 9, 5, 1 on the stress–strain diagrams in Figure 3 and Figure 4; designation (**b**) corresponds to specimen 7).

In particular, we consider the structure of the specimen fracture, which occurred after ST at room temperature (curve 11 in Figure 3 and Figure 4). It suggests (Figure 9a) that fracture followed the mechanism of separation with deep dimples in the central zone of the specimen [21]. This indicates a sufficiently high plasticity and fracture toughness of the alloy. When compared, the fractures of specimens 11 and 7 destroyed dynamically upon DNP in liquid nitrogen (Figure 9b) indicate the signs of brittle fracture. The dimples formed on the fracture surface became flatter. As the intensity of introducing impulse energy into the alloy in liquid nitrogen increases, local micro-tear-offs and elongations of adjacent sections begin to appear (Figure 9c,d). This may result from decohesion or ductile chipping. With the maximum intensity of introducing impulse energy into the alloy (Figure 9e), micro-tear-offs become more and more pronounced, causing significant delamination. Slippage, undulations at the bottom of the dimple and elongation zones become noticeable on the dimple surfaces under all the loading conditions considered. In general, dimples of ductile separation normally occur at secondary phases and on boundaries between phases. Large dimples show the signs of deformation which are represented by areas of shear. 

**Figure 10:**
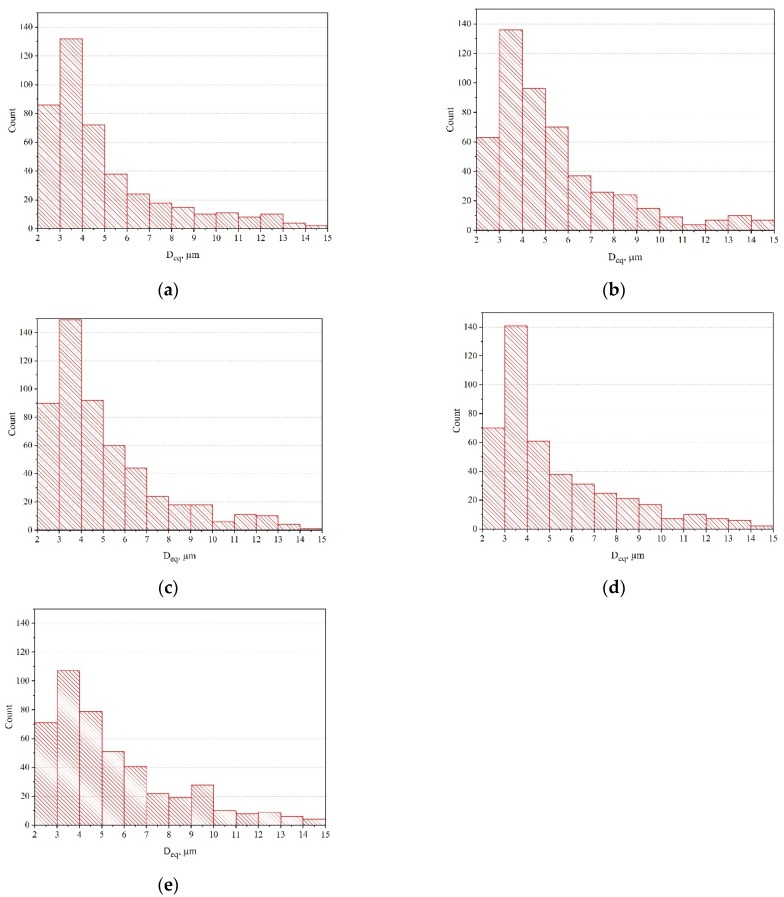
Histograms of the quantitative composition of dimples of different conventional diameters (magnification ×550) detected on the fracture surface of specimens made of titanium alloy VT23M under different loading conditions: (**a**)—1; (**b**)—5; (**c**)—7; (**d**)—9; (**e**)—11; (the numbers correspond to the numbers of specimens, for which stress–strain diagrams are shown in Figure 3 and Figure 4).

We analyze dimple dimensions on the specimen fracture. The dimensions of dimples directly depend on the number of micropore nucleation sites and the relative plasticity of the alloy matrix under specific loading conditions (Figure 10). 

Notably, the central part of all the specimens demonstrates a fairly similar fracture pattern. Therefore, it is advisable to use specimen pairs that differ significantly in deformation during failure for the analysis and comparison of conventional diameters of dimples. These are, for instance, specimen pairs 11–1, 1–5, 1–9 and, naturally, specimen pair 11–7, which were destroyed without any exposure to IOL but at different temperatures, that is, normal and cryogenic. The difference in conventional diameters of dimples is particularly noticeable when comparing specimen pairs 11–1, 11–7 and 1–5 in Figure 10. With an increase in the DNP intensity in liquid nitrogen, the number of dimples of minimum diameter increases significantly upon subsequent ST at room temperature. This is particularly noticeable when analyzing the conventional dimple diameter of specimen 11 destroyed during static tensioning at room temperature versus fractures of other specimens.

Thus, while analyzing the results of fractographic investigations into fractures of specimens, one can state that DNP of different intensities in liquid nitrogen causes damage to the alloy’s structure. When specimens from the VT23M alloy are subjected to static tensioning at normal temperature, changes occur in the nature and kinetics of the pore and microcrack accumulation. Dimples of different conventional diameters are also formed on specimen fractures [27,28]. Since this alloy appears to strengthen during deformation (see Figure 3 and Figure 4), previous damage caused to the alloy structure in liquid nitrogen begins to manifest itself upon subsequent ST. In particular, we notice changes in the descending branches of the stress–strain diagrams. In addition, the overall deformation of the alloy decreases accordingly (see Figure 3 and Figure 4). 

### 3.4. Discussion

Experimental investigations into the effect of low temperatures on changes in the mechanical properties of the VT23M alloy upon static tensioning have been conducted. Their results indicate that with a decrease in the test temperature, the ultimate strength of the material increases significantly, from σ_us_ = 1080–1180 MPa at room temperature to σ_us_~1500 MPa at a temperature of 77 K, respectively. Here, it should be emphasized that this increase also depends on the shape of the specimens tested. Thus, for instance, when testing flat specimens from the VT23M alloy, no increase occurs in the ultimate strength at a temperature of 213 K [15] compared to room temperature. Conversely, when testing cylindrical specimens, an increase in the ultimate strength of up to 7% occurs [15]. At the same time, a significant decrease (by up to 6%) in the alloy ductility was observed at a temperature of 77 K [15].

The results of mechanical tests indicate that alloy VT23M has a high resistance to DNP in liquid nitrogen (up to *ε_imp_*~0.6%). This fact makes it possible to guarantee the use of this alloy not only in the responsible structures of rocket and space technology, but also in the structures of ships that are operated in arctic climate conditions.

With an increase in *ε_imp_*, signs of embrittlement were observed, as evidenced by the results of diffractometric and fractographic research. However, this effect is not critical. The scatter of the ultimate strength values under different loading conditions upon repeated static tensioning at room temperature is within 3%, and the maximum decrease in the alloy plasticity is more significant—down to 16%.

The proposed technique for evaluating structural damage caused to the VT23M alloy based on the apparent changes in the α-phase percentage composition during loading is confirmed by the fractographic research findings.

## 4. Conclusions

The effect of DNP of different intensities in liquid nitrogen on changes in the mechanical properties of the VT23M sheet two-phase high-strength titanium alloy subjected to static tensioning at room temperature was evaluated for the first time. The ultimate strength of the VT23M alloy upon subsequent static tensioning at room temperature was shown to remain practically unchanged, provided that *ε_imp_*~0.6% after DNP in liquid nitrogen. The scatter of the ultimate strength values of the VT23M alloy was about 3% under all loading conditions. At the same time, the alloy plasticity dropped down to 16%. Therefore, we can conclude that titanium alloy VT23M shows high resistance to DNP in liquid nitrogen.

To explain a decrease in alloy plasticity after DNP, a detailed analysis of the alloy structure was performed by diffractometric and fractographic methods. Alloy VT23M became more brittle, which is due to an increased number of defects in the more brittle α-phase of the alloy. Diffractometric research findings indicate that the damageability of α-phase is 12.46% at *ε_imp_* = 0.28%. With an increase in *ε_imp_* to 0.53%, the damageability of α-phase increases to 33.21%. That is, the damageability of a more brittle α-phase increases with an increase in *ε_imp_*. The automated method for the analysis of the shape and size of dimples of ductile tearing formed during static and impact fracture of titanium alloy VT 23M was used. This made it possible to find the main difference in the alloy’s structure upon DNP of different intensities in liquid nitrogen. During tests in liquid nitrogen, *ε_imp_* increased with subsequent static stretching at room temperature. In addition, the number of dimples of minimum diameter increased significantly in the fractured specimens. This indicates the effects of embrittlement in the alloy.

The proposed method for evaluating the effect of DNP of variable intensity in liquid nitrogen on changes in the mechanical properties of alloys upon subsequent static tensioning is useful when choosing the most promising titanium alloys for manufacturing load-bearing structures of cryogenic equipment.

## Figures and Tables

**Table 1:** Mechanical properties of VT23M titanium alloy.

Titanium Alloy	Mechanical Properties		
	σ_ys_, MPa	σ_us_, MPa	δ, %
VT23M	1000–1150	1080–1180	20–21

**Table 2:** Chemical composition of VT23M titanium alloy.

Chemical Composition, % by Weight					
Fe	Cr	Mo	V	Ti	Al
0.7	1.1	2.2	4.5	86.7	4.8

## Data Availability

The original contributions presented in the study are included in the article, further inquiries can be directed to the corresponding author.
